# Factors associated with lack of postnatal care among Palestinian women: A cross-sectional study of three clinics in the West Bank

**DOI:** 10.1186/1471-2393-8-26

**Published:** 2008-07-18

**Authors:** Enas Dhaher, Rafael T Mikolajczyk, Annette E Maxwell, Alexander Krämer

**Affiliations:** 1 Department of Public Health Medicine, School of Public Health, University of Bielefeld, Bielefeld, Germany; 2 USAID funded Maram project in West Bank, Palestine; 3 School of Public Health and Jonsson Comprehensive Cancer Center, Division of Cancer Prevention and Control Research, University of California, Los Angeles, USA; 4 650 Charles Young Dr. South, A2-125 CHS, Los Angeles, CA 90095-6900, USA

## Abstract

**Background:**

Only about one-third of women in Palestine (West Bank and Gaza) obtain postpartum care. Therefore, the goal of this study was to assess factors associated with lack of postnatal care, women's reasons for not obtaining postnatal care, and their attitudes towards its importance.

**Methods:**

In early 2006, a cross-sectional survey was conducted at three clinics run by the Ministry of Health providing Mother and Child Health Care in West Bank, Palestine. A total of 264 postpartum women attending the clinics were interviewed face-to-face, using a structured questionnaire.

**Results:**

Although the majority of women considered postnatal care necessary (66.1%), only 36.6% of women obtained postnatal care. The most frequent reason for not obtaining postnatal care was that women did not feel sick and therefore did not need postnatal care (85%), followed by not having been told by their doctor to come back for postnatal care (15.5%). Based on a multivariable analysis, use of postnatal care was higher among women who had experienced problems during their delivery, had a cesarean section, or had an instrumental vaginal delivery than among women who had a spontaneous vaginal delivery. Use of postnatal care was also higher among women who delivered in a private hospital as compared to those who delivered in a public hospital. In addition, we found regional differences.

**Conclusion:**

The higher use of postnatal care among high-risk women is appropriate, but some clinically dangerous conditions can also occur in low-risk women. Future efforts should therefore focus on providing postnatal care to a larger number of low-risk women.

## Background

The postpartum period starts about an hour after the delivery of the placenta and includes the following six weeks [1]. Several studies in both high and low income countries have identified the importance of the postpartum period for acute short-term, long-term, and chronic morbidity [1-10]. Moreover, up to two thirds of maternal deaths occur after delivery [2,11]. Therefore, the World Health Organization suggests that health care should be provided at 6 hours, 6 days, 6 weeks, and 6 months post delivery, in order to ensure women's physical and mental health and well-being [1].

Despite this recommendation, seven out of ten women do not receive any postpartum care, based on Demographic and Health Surveys conducted in 30 low income countries between 1999 and 2004 [12]. Low utilization of postnatal care has been related to women's lack of knowledge about its importance, their lack of perceived need (especially if they are feeling well), their low level of education, poverty, lack of access to health care facilities that provide postnatal care, lack of appointments or recommendations from health care providers to obtain postnatal care, poor attitudes of the health care providers, or women's tendency to give priority to the health needs of their infants rather than their own [13-19].

A recent study in the West Bank – Palestine found maternal mortality ratios of 29.2 and 36.5 per 100,000 live births for the years 2000 and 2001, respectively. Of the 36 maternal deaths that were recorded, 20 (55%) occurred during the postpartum period [20]. In Palestine (West Bank and Gaza), studies conducted between 2003 and 2005 reported that only 23 to 34% of women received postpartum care [21-23].

Although the vast majority of women in Palestine (West Bank and Gaza) delivers in hospitals or health institutions such as private doctor clinics or maternity homes [22,23], the average postpartum stay in hospitals is only 24 hours [22]. As a result, women need to obtain postnatal care in community health clinics [24]. Several studies have assessed the receipt of postnatal care in Palestine [21-23], however, none of these studies have reported factors associated with its non-use. Therefore, the main goals of this study were (1) to assess factors that are associated with lack of postnatal care, and (2) to assess women's attitudes towards the importance of postnatal care and their reasons for not obtaining postnatal care.

## Methods

### Sample and instrument

We conducted a cross-sectional survey at three clinics that provide Mother and Child Health Care in the West Bank. The clinics were located in the three largest cities in the West Bank: Jenin in the north, Ramallah in the center, and Hebron in the south. They were selected from a list of clinics that was provided by the Ministry of Health (MOH) based on the following criteria: (1) the clinics should provide most of the reproductive health services (antenatal care including high-risk pregnancy, postnatal care, immunization of pregnant women against tetanus, baby immunization, and family planning); (2) they should be the referral clinic from the surrounding villages and camps; and (3) they should be sufficiently staffed and equipped (as identified by the MOH). The purpose of criteria (1) and (2) was to ensure the availability of a large number of women with diverse socio-economic backgrounds coming for a range of services from the city and surrounding areas; and the purpose of criterion (3) was to facilitate the conduct of the study as we did not want to burden an understaffed clinic with our research study. A total of 84 clinics in the West Bank met our criteria [25]. One clinic was in Jenin, two were in Ramallah, and four were in Hebron. In Ramallah and Hebron, we chose the clinics that saw the highest numbers of patients.

A questionnaire was developed on the basis of a literature review and adopted questions from previous studies including the Demographic and Health Surveys (DHS) and the national survey of Sultanate of Oman, some of which had been conducted in Arabic language [26,27]. It assessed issues related to reproductive health: postnatal care, attitudes towards domestic violence, use of family planning methods and the understanding of reproductive rights. The questionnaire was first composed in English and then translated by two independent translators into Arabic. Cases of disagreement were discussed among both translators together with the first author (ED). It was pilot tested to assess acceptability and comprehension [28] among 30 women in the Ramallah clinic. Data from the pilot tests are not included in the analyses conducted for this paper. Based on results from the pilot test, the questionnaire was slightly revised and shortened from 35 to 25–30 minutes by deleting some questions and rephrasing others. Copies of the questionnaire in English or Arabic are available from the first author upon request.

### Data collection

The three clinics were visited 6 days a week from Saturday through Thursday in Spring 2006. Two female data collectors with medical or social science backgrounds, who had experience with collecting data in health care centers, assisted the first author (ED) in the fieldwork. Data collectors attended a three-day workshop conducted by ED to ensure a common methodology at all three clinics. Women were approached after they had received their health care in the clinic. Given the mean duration clinic visit of 5 minutes and the duration of both informing the women about the study and the interview (which took between 25–30 minutes), approximately every 5th, or 6th eligible woman was interviewed. Only four women refused to participate, resulting in a response rate of 99%. The interviews took place in a private room in each clinic. About 92% of women completed the interview without any company, while 8% were accompanied by some other person(s) mainly a sister, a mother and in rare cases a mother in law. A total of 450 women (186 pregnant and 264 postpartum) completed the interview during a 12 week period. Initially, a sample size of 100 women at each clinic was planned, assuming a 75% response rate. A total sample of 300 respondents would allow estimating proportions with +/-5% accuracy for the 95% confidence interval. Given the excellent response rate in the pilot phase, the available resources were used to aim for a sample of 150 responses at each clinic, which was accomplished. This sample size provides 80% power to detect differences of 16% or more between cities. The analysis in this paper is restricted to postpartum women only.

### Dependent variables

All postpartum women who had delivered a baby within the past 15 months (N = 264) were asked whether they had obtained postnatal care any time during the first six weeks after delivery. Women who had not obtained postnatal care were asked about the reasons for that. The question was open-ended and women were able to provide multiple reasons. In the latter part of the questionnaire women were also asked about their attitudes towards postnatal care: "*In your opinion is postnatal care necessary for a woman's health?"*

### Independent Variables

The questionnaire included information about several socio-demographic variables: woman's current employment status (unemployed or employed), woman's and husband's highest level of education (< secondary or ≥ secondary school), woman's age and age at first marriage, total number of living children, and woman's self-assessed economic situation (in three categories: high, middle, low). Education of both partners was recoded into a single variable with four categories: both < secondary, wife ≥ secondary + husband < secondary, wife < secondary + husband ≥ secondary, both ≥ secondary school.

Several additional variables related to medical care were collected: delivery place, having had problems during last delivery, number of antenatal visits during the last pregnancy, and whether the woman was informed about danger signs to be monitored after delivery related to her and her baby's health before discharge from the hospital. Women who had received postnatal care were asked whether they had received advices on family planning and breast feeding.

### Statistical analysis

SPSS 12 statistical software was used to enter and analyze the data. Cross tabulation and Pearson chi-square test were used for descriptive and bivariate analyses. For the multivariable regression model the following variables were selected: location of the clinic, woman's age (in years), joint education variable, number of living children (to assess the extent of previous experiences with childbearing), number of antenatal visits during the most recent pregnancy (to assess general use of medical care), woman's age at first marriage (below or above 20 years, as a marker of more or less traditional upbringing), problems during delivery (since women who experienced problems may be more likely to receive postnatal care), delivery place (public versus private hospital, which may indicate different information received at discharge) and whether the woman was informed about danger signs for her and her baby's health before discharge. We did not include women's attitude regarding the importance of postnatal care, because attitude might be influenced by the actual behavior [29,30]. To limit the number of variables in the regression analysis, we included education only as a proxy for the socio-economic status of the women. Furthermore, we did not include receipt of information about danger signs related to the mother's or the baby's health before discharge from the hospital, because these two variables were significantly associated with place of delivery: Women who delivered at a private hospital were more likely to be informed. Reasons for not obtaining postnatal care were analyzed by grouping similar answers.

### Ethical approval

A steering committee comprised of representatives from the Palestine Ministry of Health, the United Nations Relief and Works Agency for Palestine Refugees in the Near East (UNRWA), and Juzoor Foundation, one of the largest local health non-governmental organizations, reviewed and approved the study protocol and questionnaire prior to data collection. Permission for data collection at each site was given by the Ministry of Health. Prior to the interview, each woman was asked to read and sign a consent form, which stated the purpose of the study, that participation was voluntary, and that women's responses were kept confidential.

## Results

### Description of the sample

As shown in Table 1, the majority of women (69%) in our sample had married at less than 20 years of age, had more than 1 child, were not employed outside the home, and rated their economic status as "middle class". Most women (82%) reported 6 or more antenatal visits for their last pregnancy and a spontaneous vaginal delivery without problems. Of interest is that only 17–19% of the women reported being informed about danger signs related to the baby's or the mother's health before hospital discharge. Almost two-thirds of the women considered postnatal care necessary.

**Table 1:** Characteristics of the sample and receipt of postnatal care

Factors	Total number N = 264	Total %	Received postnatal care	
			
			Yes N = 97	No N = 167
Age group				
≤24	77	29.2	33.8	66.2
25–29	85	32.2	35.3	64.7
≥30	102	38.6	40.2	59.8
Clinic location				
Jenin (Northern West Bank)	64	24.2	53.1	46.9
Ramallah (Central West Bank)	94	35.6	30.9	69.1
Hebron (Southern West Bank)	106	40.2	32.1	67.9
Wife and husband educational level				
Both<secondary (sec.)	72	27.3	27.8	72.2
Wife≥sec.+ husband<sec.	50	18.9	34.0	66.0
Wife<sec+ husband ≥ sec.	41	15.5	41.5	58.5
Both≥sec.	101	38.3	42.6	57.4
Number of living children				
1	57	21.6	40.4	59.6
2–3	113	42.8	38.9	61.1
4+	94	35.6	30.9	68.1
Age at first marriage				
<20	182	68.9	31.9	68.1
≥21	82	31.1	47.6	52.4
Woman's employment status				
Employed	42	15.5	42.9	57.1
Not employed	222	84.1	35.6	64.4
Economic status (subjective)				
High	23	8.7	47.8	52.4
Middle	197	74.6	38.6	61.4
Low	44	16.7	22.7	77.3
Number of antenatal visits during the last pregnancy				
1–5	45	18.0	28.9	71.1
6–10	133	53.2	39.1	60.9
>10	72	28.8	36.1	63.9
Delivery				
Spontaneous vaginal delivery	221	83.7	29.5	70.5
Other **	43	16.3	74.4	25.6
Delivery problems				
Yes	57	22.2	59.6	40.4
No	200	77.8	28.5	71.5
Informed about danger signs for the mother's health before discharge				
Yes	49	18.6	57.1	42.9
No	215	81.4	32.1	67.9
Informed about danger signs for the baby's health before discharge				
Yes	44	16.7	56.8	43.2
No	218	82.6	33.0	67.0
Delivery place of the last child				
Public hospital	121	46.7	31.4	68.6
Private hospital	138	53.3	40.6	59.4
Considering postnatal care necessary***				
Yes	166	66.1	46.4	53.6
No	85	33.9	18.8	81.2

** other: cesarean section or instrumental vaginal delivery

*** 13 women answered "do not know" and calculated as missing

### Postnatal care and associated factors

Among the 264 postpartum women, 97 (36.7%) obtained postnatal care. This proportion was significantly higher in Jenin than in both Ramallah and Hebron. It was also significantly higher among women who were ≥ 21 years of age at the time of their first marriage, who experienced complications during delivery, who were informed about danger signs for their own health or the health of their baby before discharge from the hospital, and who considered postnatal care necessary (Table 1), Women who had delivered in private hospitals were more likely to obtain postnatal care than women who had delivered in public hospitals (41% versus 31%), but this association was not statistically significant. Women who delivered in private hospitals reported significantly more frequently that they were informed about the danger signs for their own health or the health of their baby before discharge than women who delivered in a public hospital (data not shown). Among those who obtained postnatal care (97 women), only 30.2% received counselling regarding family planning, while about two-thirds received counselling regarding breastfeeding (data not shown).

Our multivariable logistic regression analysis (Table 2) revealed that three factors were associated with lack of postpartum care after controlling for demographic and pregnancy-related variables. Women in Ramallah (central West Bank) were significantly more likely to lack postpartum care than women in Jenin (northern West Bank). In addition, women who delivered in public hospitals, and women without problems or complications during delivery were also significantly more likely to lack postpartum care as compared to women who delivered in private hospitals and women who had problems during delivery. Woman's age and age at first marriage, woman's and husband's level of education, number of children and number of antenatal visits during the last pregnancy were not significantly associated with obtaining postpartum care.

**Table 2:** Association of socio-economic and demographic factors with non-use of postnatal care

Variable	OR (95% CI)^a^	P value
Clinic location		
Hebron (Southern West Bank)	1.7 (0.8–3.6)	0.14
Ramallah (Central West Bank)	3.6 (1.6–7.9)	0.001
Jenin (Northern West Bank)	Reference	
Woman's age	0.9 (0.9–1.0)	0.88
Both husband & wife level of education		
Both<secondary	1.9 (0.8–4.5)	0.10
wife ≥ Secondary+ husband<secondary	1.5 (0.6–3.4)	0.33
Husband ≥ Secondary + wife < secondary	0.9 (0.3–2.2)	0.87
Both ≥ Secondary	Reference	
No. of living children	1.0 (0.8–1.2)	0.84
No. of antenatal visits in last pregnancy [per visit]	0.9 (0.9–1.0)	0.72
Woman's age at first marriage		
< 20	1.6 (0.8–3.3)	0.14
≥20	Reference	
Delivery problems		
No	3.9 (2.0–7.5)	<0.001
Yes	Reference	
Delivery place		
Public hospital	1.8 (1.0–3.4)	0.045
Private hospital	Reference	

OR = odds ratio; CI = confidence interval.

^a ^adjusted for all variables in the table

### Attitudes towards postnatal care and reasons for non-use

**Figure 1:**
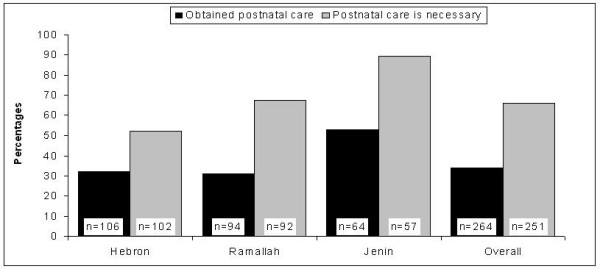
Percentages of women who obtained postnatal care (N = 264) and their attitudes towards postnatal care (N = 251; 13 women had missing values) by city.

**Figure 2:**
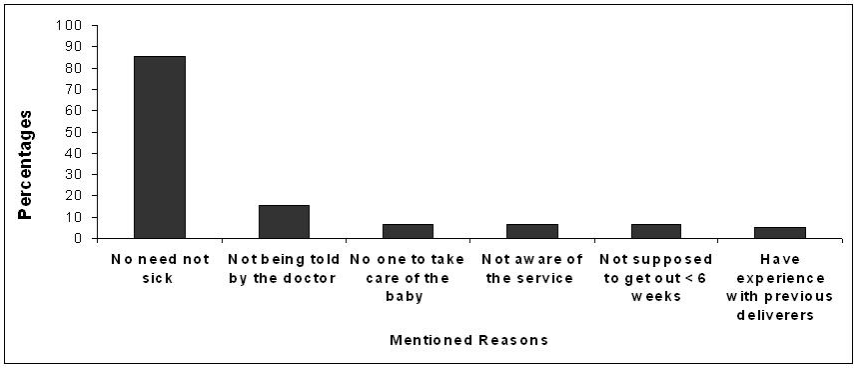
Reasons (%) for not obtaining postnatal care (N = 167 women who did not obtain postnatal care) (*percentages do not add up to 100 due to multiple answers*).

Figure 1 shows that the proportions of women who obtained postnatal care and who thought that postnatal care is necessary varied by city. Both the positive attitude towards postnatal care and the use of postnatal care were substantially higher in Jenin than in the two other cities. However, in each city, the utilization of postnatal care was much lower than the predominantly positive attitude might suggest. This prompts the question of why the majority of women do not use postnatal care despite the fact that many consider it necessary. The most frequent reason for not obtaining postnatal care was that women did not feel sick and therefore felt that they did not need postnatal care (85%), followed by not having been told by the doctor to come back for postnatal care (15.5%; multiple responses were possible). Fewer women were not aware of the service availability, had no one to take care of the children, did not want to go out before 6 weeks after delivery (which is a traditional custom in Arabic culture), or stated having experience with previous deliveries and therefore not needing additional information (Figure 2).

## Discussion

### Rate of utilization of postnatal care

This study assessed utilization of postpartum health care among women sampled at clinics in three cities of the West Bank. Consistent with two other Palestinian studies [21,22], only about one-third of the women had obtained postnatal care during the six weeks following delivery. This rate is similar to the rates found in Jordan (35%) and Egypt (41.5%) [26,27].

### Attitudes versus behavior and reasons for not obtaining postnatal care

Our data suggest that there is a notable difference between women's attitudes toward the importance of postnatal care and the actual utilization of postnatal care. This was consistently observed in all three cities (see Figure 1). One explanation for this discrepancy could be social desirability bias: Women may have overreported their belief in the importance of obtaining postnatal care so as to be viewed favorably by the interviewer. Another explanation may be that some women who believe in the importance of postnatal care cannot obtain it due to barriers, such as a lack of available services or cultural norms, including the traditional custom of not going out during the first six weeks after delivery. However, our findings suggest that this traditional custom is not a major reason for non-use of postnatal care among women in the West Bank. Most women who had not obtained postnatal care stated that they did not need it because they were not sick. This seems to be a common belief, not only in Palestine but also among women in Jordan, Lebanon, Egypt, and other countries [15,26,27,31-33]. The fact that postnatal care was perceived to be unnecessary by women who did not feel sick demonstrates these women do not recognize the importance of postnatal care for preventive health care. Some of the negative health outcomes which can occur during the puerperium may not be noticed early or initial signs might be ignored by women. Therefore, the American College of Obstetricians and Gynecologists recommends postnatal care for all women, including those who do not perceive any problems, for the purpose of general assessment of both physical and mental well-being, to rule out postpartum depression, and to discuss breastfeeding and family planning [34]. While a recent systematic review of randomized controlled trials showed that universal postpartum support to unselected women at low risk did not result in statistically significant improvements for any outcome examined, including maternal mortality, the studies included in this review were limited to high income countries [24]. Early recognition and treatment might be not as important in countries where high standards of emergency care are easily available, but may be crucial under less optimal conditions.

### Risk factors for non-use of postnatal care

Similar to other studies [13-15,17-19], our bivariate analysis revealed that women who did not use postnatal care, married at a younger age, had lower economic status, and had a spontaneous vaginal delivery without problems. Our multivariable analysis shows that after controlling for demographic characteristics, deliveries in public hospitals and without any complications were associated with non-use of postnatal care. In addition, we found regional differences: women in Ramallah were significantly more likely not to receive postnatal care than women in Jenin.

The relatively high use of postnatal care among women who had experienced problems during their delivery (60%), or had a cesarean section or instrumental vaginal delivery (75%), is reassuring because it suggests that both women and provider recognize that these factors increase the woman's risk for postnatal complications and that postnatal care is especially important for these high-risk groups [35]. Efforts should be directed to further increase the use of postnatal care among high-risk women.

The higher utilization of postnatal care by women who had delivered in a private hospital may be due to the fact that private hospitals have more resources and therefore may be more likely to provide individualized care to their patients. Our finding that women who deliver in a private hospital are significantly more likely to receive information about danger signs for their own health and the health of their baby than women who deliver in a public hospital supports this assumption. Only ten of 37 West Bank maternity hospitals are public hospitals, but they deliver almost half of all babies. In contrast, a total of 15 private hospitals deliver only 13% of all babies, with both hospital types using similar levels of staffing [36]. This may explain the high level of dissatisfaction women report with the care they receive in public hospitals, while private facilities do not garner as much criticism [37]. However, many women have no choice than to deliver in a public hospital because of low income status or lack of health insurance.

Even after controlling for the important variables "delivery with or without problems" and "private versus public hospital," we found that postnatal care was significantly more often lacking in Ramallah than in Jenin, which had the highest utilization of postnatal care of the three clinics. This is puzzling, because overall, Central West Bank; where Ramallah is located, has the highest availability of hospital services (38,668 population per hospital) while Northern West Bank, where Jenin is located, has the lowest availability of hospital services (61,548 population per hospital) [37]. This suggests that there might be other determinants of postnatal care usage that are regionally different.

### Strengths and Limitations

Strengths of this study are the systematic sampling in three different geographic regions of the West Bank and the high response rate. We did not provide monetary incentives for participation, but word about the study spread among women who were waiting to be seen at the clinics. It is possible that women participated because they were curious about the study or they were glad that someone took an interest in listening what they had to say. The interviewers were trained to respond to questions about family planning after the interview.

Limitations of this study are due to self-reported outcomes and sampling restrictions. Women may have over-reported their use of postnatal care in order to please the interviewer, especially since they were interviewed in a health care setting. However, the rate of postnatal care in our study is similar to the rates reported by two other Palestinian studies [21,22]. There may be a possible sampling restriction in this study, as all participants were recruited through Maternal and Child Care Clinics. Seemingly, women who do not obtain postnatal care would be less likely to visit these clinics and be included in this study. However, given the very high vaccination coverage in Palestine of ≥ 95% [38,39] and the fact that these clinics are the only places that offer infant vaccinations (apart from the UNRWA clinics that serve only refugee camps), most women do visit these clinics, at least to vaccinate their infants. Because most women visit these clinics and due to our sampling in three different cities, we believe that our findings reflect the experience of postpartum women in the West Bank.

## Conclusion

The higher use of postnatal care among high-risk women is appropriate, but some of the clinically dangerous conditions can also occur in low-risk women. Future efforts should therefore focus on also increasing participation in postnatal care among low- risk women.

## Competing interests

The authors declare that they have no competing interests.

## Authors' contributions

As part of her dissertation, ED designed the study, performed and supervised data collection, conducted the analysis, and drafted the manuscript. RTM and AEM, participated in designing the study and in developing the questionnaire, supervised the analysis, and wrote the final version of the manuscript. AK supervised the development of the study design, provided comments for the study questionnaire and participated in writing the final version of the manuscript. All authors read and approved the final manuscript.

## Pre-publication history

The pre-publication history for this paper can be accessed here:


